# Dosimetric Accuracy of MR-Guided Online Adaptive Planning for Nasopharyngeal Carcinoma Radiotherapy on 1.5 T MR-Linac

**DOI:** 10.3389/fonc.2022.858076

**Published:** 2022-04-07

**Authors:** Shouliang Ding, Hongdong Liu, Yongbao Li, Bin Wang, Rui Li, Xiaoyan Huang

**Affiliations:** State Key Laboratory of Oncology in South China, Collaborative Innovation Center for Cancer Medicine, Sun Yat-sen University Cancer Center, Guangzhou, China

**Keywords:** online adaptive radiotherapy, synthetic CT, bulk ED assignment, MR-Linac, NPC

## Abstract

**Purpose:**

The aim of this study is to evaluate the dose accuracy of bulk relative electron density (rED) approach for application in 1.5 T MR-Linac and assess the reliability of this approach in the case of online adaptive MR-guided radiotherapy for nasopharyngeal carcinoma (NPC) patients.

**Methods:**

Ten NPC patients formerly treated on conventional linac were included in this study, with their original planning CT and MRI collected. For each patient, structures such as the targets, organs at risk, bone, and air regions were delineated on the original CT in the Monaco system (v5.40.02). To simulate the online adaptive workflow, firstly all contours were transferred to MRI from the original CT using rigid registration in the Monaco system. Based on the structures, three different types of synthetic CT (sCT) were generated from MRI using the bulk rED assignment approach: the sCT_ICRU_ uses the rED values recommended by ICRU46, the sCT_tailor_ uses the patient-specific mean rED values, and the sCT_Homogeneity_ uses homogeneous water equivalent values. The same treatment plan was calculated on the three sCTs and the original CT. Dose calculation accuracy was investigated in terms of gamma analysis, point dose comparison, and dose volume histogram (DVH) parameters.

**Results:**

Good agreement of dose distribution was observed between sCT_tailor_ and the original CT, with a gamma passing rate (3%/3 mm) of 97.81% ± 1.06%, higher than that of sCT_ICRU_ (94.27% ± 1.48%, *p* = 0.005) and sCT_Homogeneity_ (96.50% ± 1.02%, *p* = 0.005). For stricter criteria 1%/1 mm, gamma passing rates for plans on sCT_tailor_, sCT_ICRU_, and sCT_Homogeneity_ were 86.79% ± 4.31%, 79.81% ± 3.63%, and 77.56% ± 4.64%, respectively. The mean point dose difference in PTV_nx_ between sCT_tailor_ and planning CT was −0.14% ± 1.44%, much lower than that calculated on sCT_ICRU_ (−8.77% ± 2.33%) and sCT_Homogeneity_ (1.65% ± 2.57%), all with *p* < 0.05. The DVH differences for the plan based on sCT_tailor_ were much smaller than sCT_ICRU_ and sCT_Homogeneity_.

**Conclusions:**

The bulk rED-assigned sCT by adopting the patient-specific rED values can achieve a clinically acceptable level of dose calculation accuracy in the presence of a 1.5 T magnetic field, making it suitable for online adaptive MR-guided radiotherapy for NPC patients.

## Introduction

Nasopharyngeal carcinoma (NPC), originated from the nasopharyngeal mucosal lining, is a malignancy caused by multiple factors involving environmental factors, genetic variants, and Epstein–Barr virus (EBV) infection (1, 2). Due to its high sensitivity to ionizing radiation and deep-seated anatomic location, radiotherapy has been established as the mainstay treatment modality since 1965 (2). In the last decade, intensity modulated radiotherapy (IMRT) has been a widely used technique in the treatment of NPC (3). IMRT can modulate both the intensity and shape of individual beams to achieve an optimal dose distribution to tumor area. A more conformal dose distribution enables IMRT to minimize the dose delivery to organs at risk (OARs), including the optic chiasm, spinal cord and brain stem (4–6). However, the IMRT treatment process usually lasts for several weeks. During the treatment process, the tumors or normal organ volume shrinkage and weight loss are commonplace in NPC patients receiving radiotherapy (7, 8). Considering that some important normal organs (such as the brain stem, spinal cord, optic chiasm, and optic nerve) in the NPC site are very close to the tumor, these OARs may move into high-dose regions when the volume of tumor is shrunk (8, 9).

Recently, adaptive radiotherapy (ART) has aroused the attention of many oncologists, which offers the possibility to correct these variations in dosing regions (2). ART means acquisition of a new set of images at some point during treatment, alteration of the radiotherapy treatment plan parameters based on new imaging findings, and delivery of the new plan for the remainder of the treatment (10). Several studies have reported that NPC patients can benefit from ART during the radiotherapy treatment (11–13). For instance, Chen et al. compared the survival outcomes in patients with or without ART, and reported that the 2-year locoregional relapse-free survival for patients treated by ART or not were 88% and 79%, respectively (*p* = 0.01) (11). These studies about ART for NPC patients are all based on computed tomography (CT) or cone-beam CT (CBCT) images. CT images, the clinical standard method for acquiring electron density, are the best choice for adaptive re-planning. However, acquiring new CT images increases the workload in busy clinical departments and at the cost of extra radiation to patients. As to the CBCT technique, it is also not applicable due to the relatively low image quality and the inability to directly use its Hounsfield Unit (HU) values for dose calculations (14–16).

In recent years, magnetic resonance imaging (MRI) has been widely applied in radiation therapy workflow. MRI can provide higher soft-tissue resolution and superior target volume delineation than CT, especially for NPC, which may appear larger and more irregularly shaped on MRI (17). Additionally, MRI has been found to improve evaluation of the extent of primary nasopharyngeal tumor and retropharyngeal lymph node metastasis (1, 18). Recently, the integrated MRI with linear accelerator delivery systems (Elekta 1.5 T Unity MR-Linac, ViewRay Inc 0.35 T MRIdian MR-Linac and ^60^Co system) have been clinically available and the potential advantages of MRI-guided radiotherapy have been explored (19–21). The MR-guided radiotherapy systems allow the creation of the radiotherapy treatment plan directly on the daily MRI acquired by the on-board scanner, considering the actual patient anatomy and so addressing the inter-fraction organ variability (22). MR-Linac brings the online ART into reality and also provides a simple and convenient solution. However, a critical challenge in current MR-Linac is that MRI requires the assignment of a relative electron density (rED) map to allow for dose calculation (23).

Different approaches have been developed to assign rED map to MRI for dose calculation in online ART, such as bulk density assignment, deformable registration-based technique, voxel-based substitution, atlas-based methods, and deep learning-based methods (24–26). However, the synthetic CT (sCT) generated by bulk rED assignment strategy is currently the only one available in Unity MR-Linac clinical workflow (22, 27, 28). The mean rED values in sCT are derived from the delineated volumes in the registered original CT images. This approach can be useful for the online adaptive procedures, as it allows a fast update of the rED map, overcoming the need to manually override the errors due to the deformable registration.

Several studies have investigated the dosimetric feasibility of using the bulk approach to generate sCT for abdominal, pelvic, and cervix patients (22, 27, 29). Prior et al. have evaluated such method in the pancreatic and prostate cases under 1.5 T magnetic field, demonstrating that the uniform rED assignment combined with the presence of magnetic field can result in differences up to 5%–9% in the DVH parameters, while the same procedure without magnetic field leads to differences of 3%–5% (29). Cusumano et al. also reported that the sCT generated using bulk rED assignment for pelvic and abdominal sites can guarantee a high level of dose calculation accuracy in the presence of 0.35 T magnetic field, and the adoption of patient-specific bulk rED values can improve the dose accuracy in all cases (22).

Compared with pelvic and abdominal cancer, NPC involves a variety of OARs and contains different tissue density areas such as the bones, cavity, and soft tissues. The presence of a longitudinal magnetic field inevitably affects radiation dose distribution. Particularly at tissue–air boundaries, significant dose changes are observed due to the electron return effect (ERE) as the generated secondary electrons can be forced back into the tissue by the Lorentz forces (30, 31). The NPC patients have a more complicated anatomy and more cavities; therefore, the impact of magnetic field in dose calculation should be more significant. Several studies have investigated the feasibility of bulk density assignment in MRI for NPC IMRT treatment planning (32, 33). For instance, Young et al. investigated the dosimetric and optimization errors due to differences in rED values when converting MRI into sCT for dose calculation, and reported that density correction using a bulk density approach achieves dose calculation uncertainties within 3% (33). Chin et al. investigated the feasibility and limitations of bulk density assignment in MRI for head and neck IMRT treatment planning (32). However, all the published studies did not account for the impact of magnetic field in dose calculation. Actually, in MR-guided radiotherapy, the magnetic field is always on, even during the radiation delivery, as demonstrated by several experiences (34, 35). As a result, the clinical feasibility of the bulk rED assignment approach has to be evaluated considering the presence of magnetic field.

Therefore, in this study, we aim to investigate the dose calculation accuracy of using the bulk rED-assigned sCT in the presence of a 1.5 T magnetic field, and evaluate the reliability of this approach in the case of online adaptive MR-guided radiotherapy for NPC patients.

## Materials and Methods

### Data Acquisition

A total of 10 NPC patients formerly treated with IMRT on conventional linac from June 2020 to December 2020 were retrospectively selected and investigated in this study. All patients had pathological confirmed, poorly differentiated squamous cell carcinoma. The median age of the patient cohort is 41 years old (ranging from 30 to 75). Four female and six male patients were included in this cohort. The patients who have dental implants in place were excluded.

The original planning CT and T2 MRI datasets were utilized in the study. CT simulations of the ten patients were performed on a large bore CT scanner (Philips Brilliance™, Netherlands) in supine position (patient-specific polyurethane foam immobilization devices and head–neck–shoulder immobilization mask), with a 140-kVp voltage, a field of view (FOV) of 80 cm, a uniform slice thickness of 0.3 cm, and a pitch of 1:1. The scan range was from the top of the head to 2 cm below the clavicle. Each patient also underwent MR scanning on an MRI simulator (Philips Ingenia 3.0 T, Netherlands) using the same scan range and the same immobilization. Both the MR and CT images for each patient were acquired at the same day, typically within an interval of 2 h. After scanning, the CT and MRI acquired were then transferred to the Unity MR-Linac (Elekta, AB, Stockholm, Sweden)-specific treatment planning system (TPS) Monaco (v5.40.02) for structure delineation and treatment planning.

### Delineations of Target Volume and OARs

Based on the CT and MR images, targets and OARs were delineated in the Monaco system by a senior radiation oncologist specialized in NPC. The delineations were performed in accordance with the guidelines in ICRU report 62 (36) and ICRU report 50 (37). The target volumes delineated contain the gross tumor volume in the nasopharynx (GTV_nx_), the nodal target volume in the neck (GTV_nd_), the high-risk clinical target volume (CTV_1_), and the preventive clinical target volume (CTV_2_). Based on the above delineated target volumes, the planning target volumes (PTVs) were generated through margin expansion to account for positioning errors, defined as PTV_nx_, PTV_nd_, PTV_1_, and PTV_2_, respectively. OARs were delineated according to the ICRU report 83 (38). Structures such as the spinal cord, brain stem, optic nerve, lens, optic chiasm, eyes, parotids, and temporal lobe were included. Additionally, to improve the accuracy of sCT conversion, the bone and air regions were also contoured for each case. Threshold segmentation with manual editing was performed on the CT to delineate bone (Hounsfield number HU > 250) and air (HU < −300) regions.

### Synthetic CTs Generation

In the Unity MR-Linac online radiotherapy workflow (28), daily MRI was acquired on the Unity system and automatically sent to the online Monaco TPS. Then, an automatic rigid registration was performed between the reference planning CT and the MRI. Manual adjustment may be needed in this step if necessary. Two different plan adaption strategies can be chosen depending on the clinical situation on the day. The first option was “Adapt to Position” (ATP), where the shape and weight of beam segments in the reference plan (CT based) were adjusted to match the current position of targets and OARs based on rigid registration. The second option was “Adapt to Shape” (ATS), where a new plan was created on the daily MRI to account for the anatomy on the day. The contours on reference CT will be propagated to the MRI using rigid or deformable registration, followed by manual editing if necessary. The sCT was generated by assigning mean rED values to the contours on MRI, where mean rED values were derived from planning CT. In ATP, the dose calculation on the day was performed on the initial reference planning CT, while the MRI on the day was only used for dose calculation in ATS.

In this study, to simulate the workflow of ATS in Unity MR-Linac, paired MR/CT images for each patient were firstly aligned using a rigid registration algorithm, with manual adjustments and careful inspection by the oncologist. In order to fairly compare and evaluate the dose and DVH difference between sCT and planning CT, the structures of targets, OARs, bone, and air were then copied from the original planning CT to the MRI. The sCT was generated from MRI by using bulk rED assignment approach, based on the mean rED values of the delineated region of interests (ROIs) derived from planning CT. All the steps to generate sCT were performed in the Unity MR-Linac-specific Monaco system.

To investigate the strategy and accuracy of bulk rED assignment, three different types of sCT images were generated by assigning different bulk rED values to the levels segmented on the original CT. The datasets were density corrected in various ways to produce image datasets with different rED features as per **Table 1**. Two sets of bulk density values were used for air and bone, respectively. The first synthetic CT (sCT_ICRU_) was created using the rED values recommended by ICRU report 46 (39) for cranium bone and air regions (bone = 1.61, air = 0.001). The remaining tissues of the patient data (such as spinal cord, brain stem, optic nerve, lens, optic chiasm, eyes, parotids, and temporal lobe) were set to patient-specific mean rED values of the delineated ROIs in the original CT.

**Table 1:** Datasets and description used in the study.

Dataset	Description
Original CT	The original CT dataset—gold standard electron density data.
sCT_homogeneity_	Entire patient dataset rED changed to a water equivalent value of 1.
sCT_ICRU_	The rED values of air and bone were recommended by ICRU Report 46 (air = 0.001, bone = 1.61, the remaining tissue of the patient data was set to mean rED values of the delineated region of interest in the original CT.
sCT_tailor_	The rED values of air, bone, and other delineated region of interest were used as patient-specific mean rED values in the original CT.

The second synthetic CT (sCT_tailor_) was generated by using the patient-specific mean rED values in original CT for air, bone, and other delineated ROIs, which is also the standard method recommended by the vendor to use in clinical practice. For comparison, the third synthetic CT (sCT_Homogeneity_) was created using a water equivalent value of 1 for the entire body.

### Treatment Plan Generation

A total of four IMRT treatment plans were calculated for each patient using the Unity MR-Linac specific TPS Monaco (v5.40.02), with consideration of the effect of the 1.5 T magnetic field by employing a graphic processing unit (GPU)-based Monte Carlo dose calculation platform (GPUMCD) (40).

The prescription doses for all the ten patients were as follows: PTV_nx_, 70 Gy; PTV_nd_, 70 Gy; PTV_1_, 60 Gy; and PTV_2_, 54 Gy in 33 fractions. All plans required that the prescription dose coverage of the target volume PTVs should be ≥95% and the maximum dose should not exceed 110% of the prescription dose. OAR dose constraints were based on ICRU report 83 [37], RTOG protocol 0615 (41), and the international guidelines on the dose prioritization and acceptance criteria in radiotherapy planning for NPC (42).

For each case, a nine-equidistant-co-planar-field (0°, 40°, 80°, 110°, 160°, 200°, 250°, 280°, and 320°) IMRT plan was firstly created on the original CT dataset and considered as the reference plan. This reference plan was then replicated to the three sets of sCT images (sCT_ICRU_, sCT_Tailor_, and sCT_Homogeneity_), respectively, with only final dose recalculation, no optimization, or any modification. Reference plans were designed with “step-and-shoot”, which is currently the only available IMRT technique in the Unity MR-Linac system. All the plans (including the reference and recalculated plans) were calculated based on the Unity machine model with 7 MV flattening filter free (FFF) photons, using 0.2cm grid spacing and a 2% statistical uncertainty per control point. Other parameters pertaining to the IMRT plans such as segment area, segment width, and number of segments per plan are outlined in **Table 2**.

**Table 2:** Calculation and segmentations for the IMRT plans.

Plan parameters	MR-Linac IMRT
Energy	7 MV FFF
Algorithm	GPUMCD
IMRT technique	Step-and-shoot
Grid spacing (cm)	0.2
Statistical uncertainty (%) per control point	2
Minimum segment area (cm^2^)	4
Minimum segment width (cm)	0.6
Minimum MU/segment	5
Maximum # segments per plan	100

### Dosimetric Evaluation

**Figure 1:**
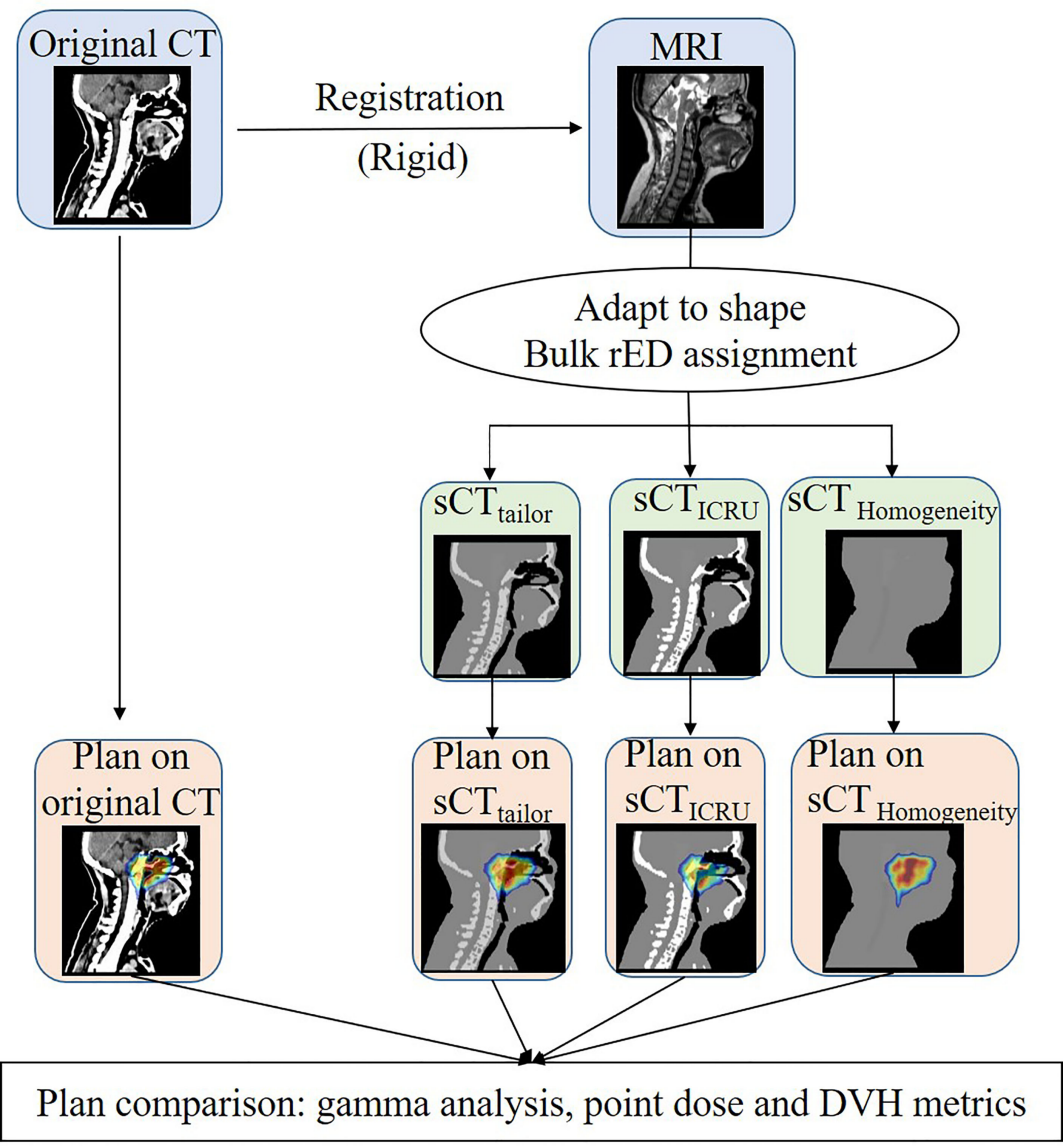
Workflow used to generate the three sCT image and to assess the dose calculation accuracy of the bulk approach with respect to sCT_ICRU_.

The recalculated dose distributions obtained on the sCT images were compared to the reference plan on the original CT. The workflow about evaluating the dose calculation accuracy of sCT is outlined in **Figure 1**. Evaluations in terms of gamma analysis, point dose comparison, and dose volume histogram (DVH) parameters were performed. Gamma analysis was performed to evaluate the dose differences in both absolute value and spatial distribution, considering the following tolerance criteria: 1%/1 mm, 3%/3 mm, and 10% dose threshold. In addition, the point dose in the PTV high-dose region was also compared. The high-dose point was selected at the center of PTV_nx_. The dose calculation accuracy was furthermore investigated by comparing the DVH parameters, such as D_mean_ (the mean dose), V_100%_ (% PTV volume covered by 100% of prescription dose), D_98%_ (the dose covered by 98% of PTV volume), and D_2%_ (the dose covered by 2% of PTV volume) for the target dose, while the D_max_ (the maximum dose), D_1cc_ (the maximum dose covering 1 cm^3^ volume), and D_mean_ for OARs.

A Wilcoxon signed rank test was performed to evaluate the statistical significance of the differences by using IBM SPSS (v25) statistical software (IBM Corporation, Armonk, NY, USA). *p <* 0.05 was considered statistically significant.

## Results

**Table 3** shows the gamma passing rates (GPRs) of recalculated dose distributions based on different sCTs, compared with the reference dose on planning CT; both 1%/1 mm and 3%/3 mm criteria were investigated. For both criteria, GPR results of plans on sCT_tailor_ are always higher than that on sCT_ICRU_ and sCT_Homogeneity_. With stricter criteria, the GPR differences among three sCT sets are more distinguishable. On average, the 1%/1 mm GPR for plans on sCT_tailor_, sCT_ICRU_ and sCT_Homogeneity_ were 86.79% ± 4.31%, 79.81% ± 3.63%, and 77.56% ± 4.64% (mean ± SD), respectively. While for criteria with 3%/3 mm, GPR for plans on sCT_tailor_, sCT_ICRU_, and sCT_Homogeneity_ were 97.81% ± 1.06%, 94.27% ± 1.48%, and 96.50% ± 1.02%, respectively. A statistically significant improvement in GPR was observed using sCT_tailor_ in the case of 1%/1 mm (*p* = 0.005) and 3%/3 mm (*p* = 0.005).

**Table 3:** Gamma passing rate comparison for 10 patient plans on sCT_tailor_, sCT_ICRU_, and sCT_Homogeneity_ (1%/1 mm, 3%/3 mm).

Patient	1%/1 mm (%)			3%/3 mm (%)		
	sCT_tailor_ vs. CT	sCT_ICRU_ vs. CT	sCT _Homogeneity_ vs. CT	sCT_tailor_ vs. CT	sCT_ICRU_ vs. CT	sCT _Homogeneity_ vs. CT
1	78.53	73.68	72.80	96.07	94.28	94.85
2	89.78	80.91	75.87	98.69	92.91	96.58
3	84.57	78.19	73.70	98.33	94.35	97.39
4	90.65	83.23	83.26	99.45	97.21	97.68
5	80.91	73.99	73.30	96.82	92.83	95.18
6	90.87	83.93	84.49	98.42	95.57	97.95
7	89.02	81.60	76.11	97.93	95.44	96.28
8	85.23	78.76	78.17	96.75	93.77	95.82
9	88.86	82.76	83.87	97.13	93.97	96.65
10	89.48	81.02	74.00	98.46	92.35	96.65
Mean (SD)	86.79 (4.31)	79.81 (3.63)	77.56 (4.64)	97.81 (1.06)	94.27 (1.48)	96.50 (1.02)

The point dose comparison in the PTV_nx_ high-dose region for all cases is presented in **Table 4**. The point dose differences for the sCT_tailor_, sCT_ICRU_, and sCT_Homogeneity_ were −0.14% ± 1.44%, −8.77% ± 2.33%, and 1.65% ± 2.57%, respectively, when compared to the original CT. Obviously, the sCT_tailor_ using patient-specific mean rED behaved with higher point dose accuracy.

**Table 4:** Point dose comparison for different sCT based plans.

Patient	sCT_tailor_ – CT (%)	sCT_ICRU_ – CT (%)	sCT _Homogeneity_ – CT (%)
1	−0.33	−6.13	3.41
2	−1.28	−6.26	−1.47
3	−1.33	−8.38	1.45
4	0.15	−9.94	4.88
5	0.95	−9.27	−1.39
6	3.42	−5.41	5.74
7	−0.65	−7.23	2.18
8	−0.45	−11.37	1.23
9	0.16	−11.97	−2.24
10	−2.02	−11.73	2.73
Mean (SD)	−0.14 (1.44)	−8.77 (2.33)	1.65 (2.57)

**Figure 2:**
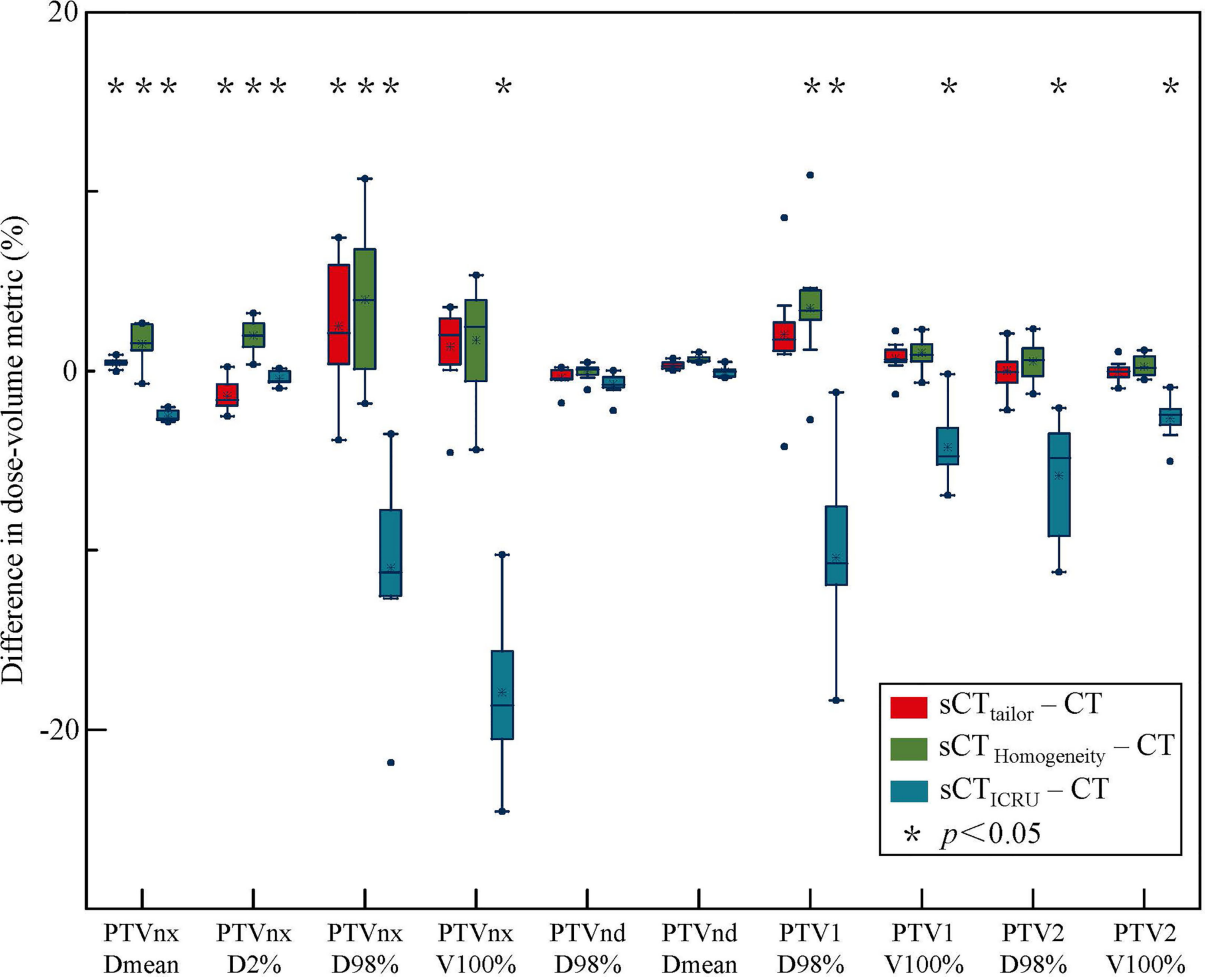
Box-plot analysis related to the dose differences of sCT_tailor_, sCT_ICRU_ and sCT_Homogeneity_ respect to the original CT for different DVH parameters related to PTV coverage. A * indicates a significance of *p* < 0.05.

**Figure 3:**
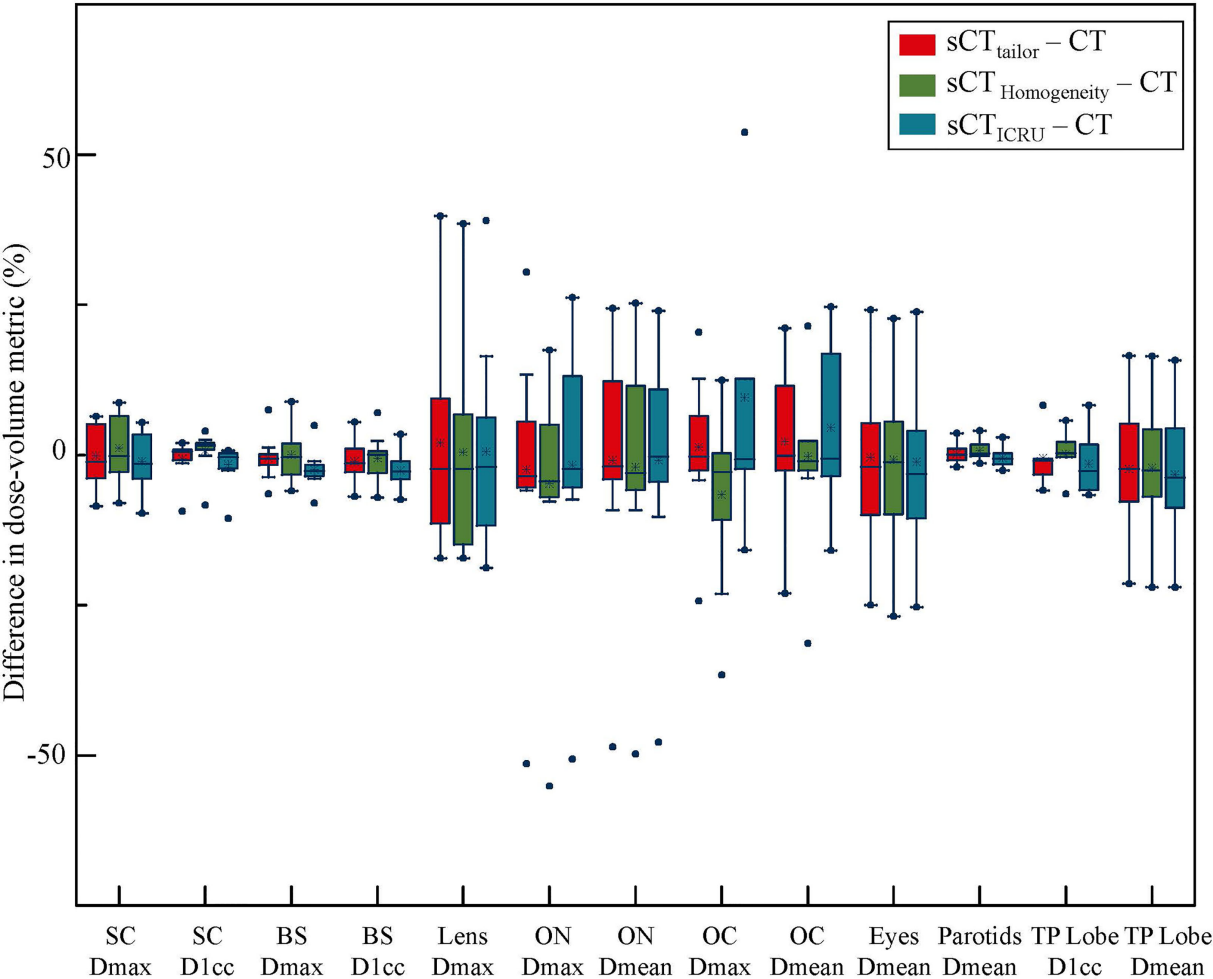
Box-plot analysis related to the dose differences of sCT_tailor_, sCT_ICRU_, and sCT_Homogeneity_ with respect to the original CT for different DVH parameters related to OAR sparing. The dot marks the outlying values.

**Figures 2**, **3** report the box plot analysis regarding the differences of the DVH parameters between the three sCT images and the original CT, separately for PTV (**Figure 2**) and OARs (**Figure 3**). For PTV coverage, the differences of the DVH parameters calculated on sCT_tailor_ are much lower than those calculated on sCT_ICRU_ and sCT_Homogeneity_. Statistically significant (*p <* 0.05) differences for PTV_nx_, PTV_1_, and PTV_2_ are observed. The mean differences in estimating the V_100%_ of PTV_nx_, PTV_1_, and PTV_2_ were found with 1.3%, 0.7%, and −0.1% for using sCT_tailor_, 1.7%, 1.0%, and 0.3% for using sCT_Homogeneity_, and 17.9%, −4.3%, and −2.6% for using sCT_ICRU_. The difference of PTV_nd_ coverage was found with no statistical significance. For OARs, the differences on the DVH parameters calculated on the three sCT images were observed with a similar level, also with no statistical significance.

**Figure 4:**
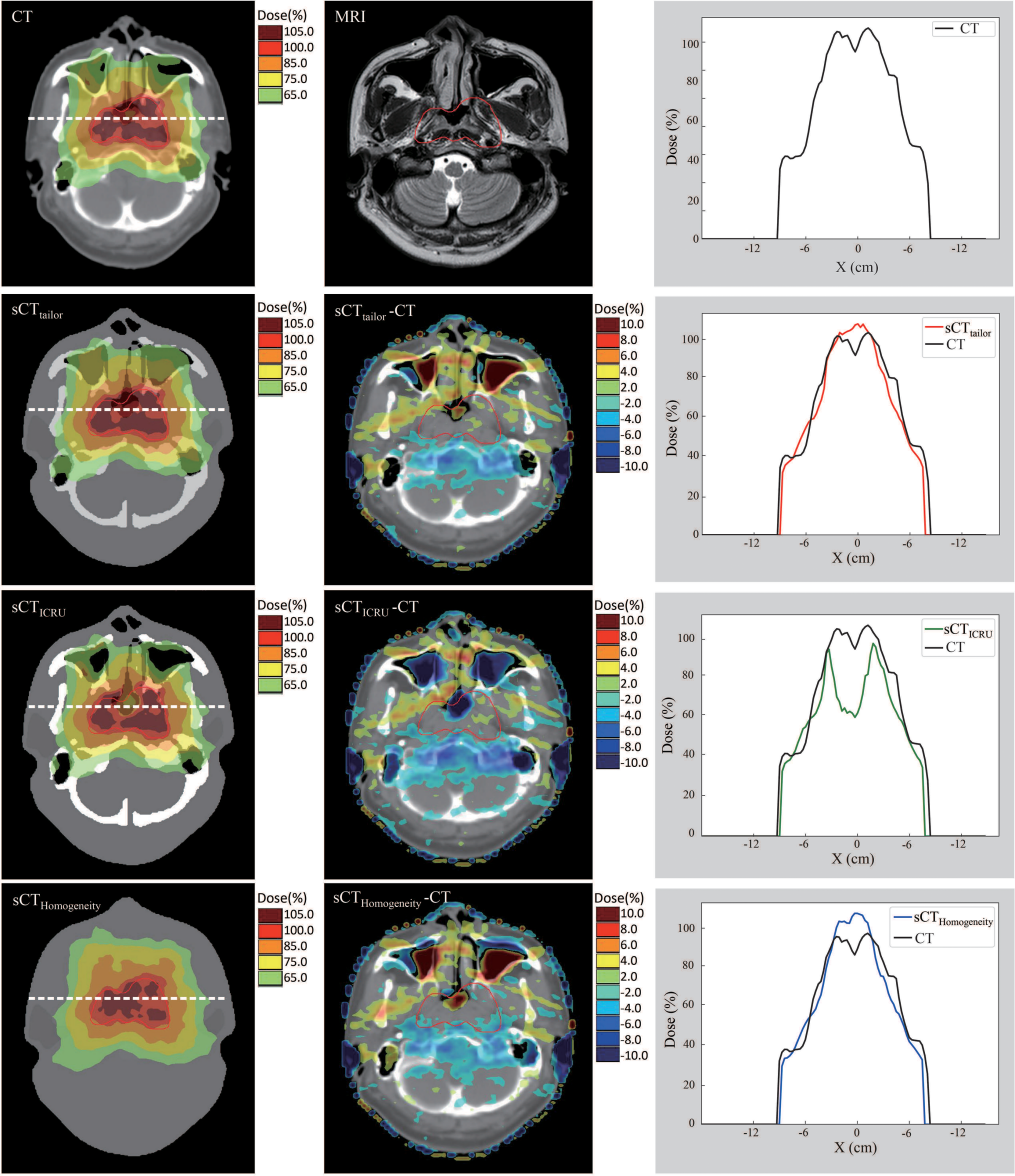
Dose comparison of plans on sCT_tailor_, sCT_ICRU_, and sCT_Homogeneity_ with respect to the original CT for dose difference maps and line profile for one NPC case.

**Figure 5:**
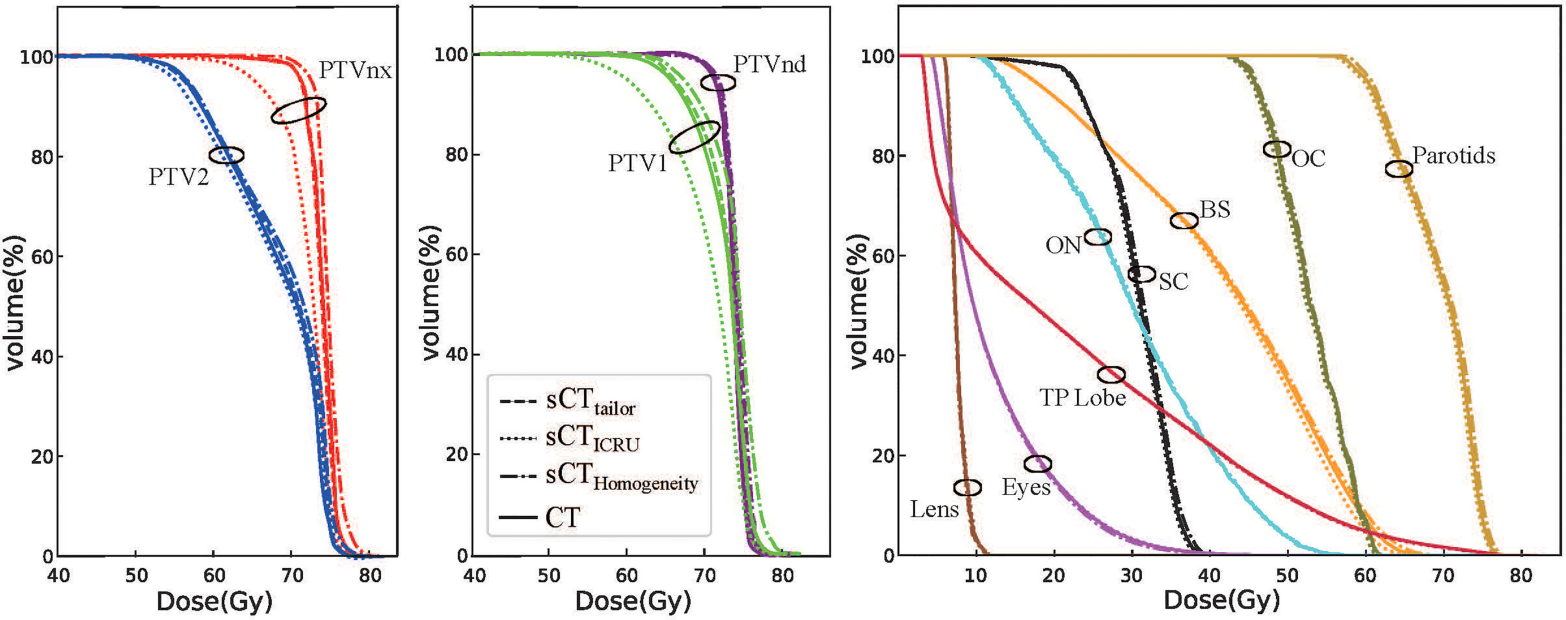
DVH comparison of plans on sCT_tailor_, sCT_ICRU_, and sCT_Homogeneity_ with respect to the original CT for one NPC case.

**Figures 4**, **5** show a dosimetric and DVH comparison among plans on the three sCT images for one representative NPC case. As shown in the dose difference map in **Figure 4**, a relatively large difference was observed at the air–tissue interface, air cavity, and bony areas. The dose profiles in **Figure 4** clearly described the dose difference between plans on different sCT images and original CT. The white line in the left subfigure shows the profile position, which is selected as traversing the bony area, air–tissue interface, and air cavity, so that the dose difference can be clearly distinguished. Comparisons of the DVH curves for this NPC case are also provided, as shown in **Figure 5**. DVH results of PTV_nx_ and PTV_1_ have only minor differences when comparing the dose on sCT_tailor_ with that on the original CT, while relatively large variations were observed on sCT_ICRU_ and sCT_Homogeneity_, except that the DVH curves for PTV_nd_, PTV_2_, and all the OARs are in a similar level, with no significant difference.

## Discussion

MR-guided radiotherapy has found increasing application over recent years with the clinical introduction of systems such as the Elekta and ViewRay MR-guided radiotherapy system. Several literatures have reported MR-guided online adaptive workflow and have shown that daily adaptive radiotherapy is feasible. For instance, Intven et al. described the implementation and initial experience of MR-guided radiotherapy on the 1.5 T Unity MR-Linac and evaluated patient compliance, treatment time, and target coverage (43). They demonstrated that MR-guided radiotherapy using daily full online recontouring and replanning on 1.5 T MR-Linac for rectal cancer is feasible and currently takes about 48 min per fraction. de Muinck Keizer et al. investigated prostate intrafraction motions during entire MR-guided radiotherapy sessions on 1.5 T MR-Linac and showed that high-quality 3D cine-MR imaging and prostate tracking during radiotherapy is feasible with beam-on (44). Additionally, Padgett et al. assessed the online adaptive MR-guided stereotactic body radiotherapy (SBRT) of liver cancers on the 0.35 T ^60^Co-based MRgRT system and demonstrated that daily planning re-optimization resulted in better target conformality, coverage, and OAR sparing (45). Schaule et al. described intrafractional stability of MR-guided online adaptive SBRT for prostate cancer on ViewRay 0.35 T MR-Linac, and reported that the dosimetric benefit of MR-guided online adaptation for prostate SBRT was robust over 45 min (46).

MRI-based radiotherapy treatment planning is a necessary step to achieve adaptive radiotherapy with the ATS workflow in Unity MR-Linac, in which the daily MRI will be registered and re-contoured for adapting the treatment plan. MRI requires the assignment of a rED map as sCT to allow for dose calculation. Despite the fact that various techniques, including voxel, atlas, and machine learning methods, have been developed to generate the sCT dataset from MRI, the bulk rED-based sCT generation method is currently the only one available adopted by Unity-specific Monaco TPS. Electron density correction can influence the dose calculation accuracy and directly affect the patient clinical treatment, as this may alter the delivery of correct dose to patients (33). Furthermore, the dose calculation error introduced by sCTs may be even worse for NPC patients in the presence of the magnetic field, considering the complicated anatomy, and contains highly heterogeneous tissue areas, such as air cavities and bony regions. To our knowledge, this study is the first to investigate the dosimetric accuracy of MRI-based planning for NPC patients treated on the 1.5 T MR-Linac.

In MRI-based radiotherapy planning ATS workflow, bony region and air cavity should be delineated and assigned with appropriate rED in order to achieve accurate dose calculation, especially for NPC cases. For instance, both Alexander et al. (32) and Young et al. (33) have demonstrated dosimetric accuracy of rED assignment for bony region and air cavity compared with uniform water assignment for head and neck patients. It is also verified in our study that visible dosimetric differences between plans based on sCT_Homogeneity_ and original CT were observed. When the entire volume inside a patient’s body is assigned with a water equivalent value of 1, the sCT_Homogeneity_ cannot realistically reflect the dose distribution in air cavity and bony region, as well as the ERE at air–tissue interfaces under a 1.5 T magnetic field. Special attention should be paid to contour the cranium bone using threshold method in the Unity-specific Monaco system, and since it cannot recognize the ring structure, manual modifications are typically required.

The rED values used in some previous studies about MRI-only treatment planning were derived from ICRU report 46 (29, 33). They are collected from particular population-averaged values of healthy tissue as stated in the ICRU report. Nonetheless, based on our study, it should be used cautiously. Several literatures have reported that the dosimertric accuracy of sCT generated by bulk approach could be improved by replacing the rED values recommended by ICRU report 46 with population-averaged values (47, 48). In our study, the idea of investigating a tailored rED approach for the sCT generation was also inspired by their results. In ICRU report 46, the rED values of air regions and cranium bone are 0.001 and 1.61, respectively, while in our study, the tailored rED values of air regions were 0.196–0.327, and the values of cranium bone were 1.280–1.362 for NPC patients.

For all cases in this study, whether in criteria 3%/3 mm or 1%/1 mm, the GPRs of sCT_tailor_-based plans were always higher than that of sCT_ICRU_-based plans. When using stricter criteria of 1%/1 mm, the GPR difference between plans on sCT_tailor_ and sCT_ICRU_ became more distinguishable. The global GPR results for sCT_tailor_-based plans can be higher than 95% under 3%/3 mm criteria, while the sCT_ICRU_-based plans cannot. In addition, the point dose comparison in the PTV_nx_ high-dose region showed that sCT_tailor_ provides dose calculation error within 3% when compared with the original CT-based reference treatment plans. In contrast, the dose calculation error for sCT_ICRU_ was about 8%. Maximum dose difference of center point in PTV_nx_ was observed in Patient 9, up to 11.97%. This is higher than what was reported in previous literatures (33, 49). The reason is mainly that the selected center point is located at the tissue–air interface. When in the presence of a 1.5 T magnetic field, remarkable dose escalation appeared at the tissue–air interface due to secondary electrons forced back into the tissue by the Lorentz force (30), which was not considered in previous studies. In the original CT, the rED values at tissue–air interfaces were approximately 0.2 due to scattering and interpolation. When the air cavity was assigned with tailored rED (0.196–0.327), the rED values at tissue–air interfaces in sCT_tailor_ were closer to that in the original CT, while the rED value of 0.001 used in sCT_ICRU_ apparently underestimated the attenuations at tissue–air interfaces. Using the tailored rED values in the bulk approach can reflect the ERE and dose at tissue–air interfaces in a more realistic manner.

The differences in the DVH parameters also showed that using sCT_tailor_ is more accurate when compared with sCT_ICRU_ (**Figures 2**, **3**). The coverage differences of PTV_nx_, PTV_1_, and PTV_2_ are statistically significant (*p <* 0.05), while the differences of PTV_nd_ coverage and OARs are not. The reason can be attributed to the presence of air cavity in PTV_nx_, PTV_1_, and PTV_2_, while the PTV_nd_ and OARs have less air regions. Accounting for air cavity assigned with tailored rED values can reduce these differences significantly. The comparison of DVH curves also demonstrated this point (**Figure 5**). The dose difference maps and dose profile (**Figure 4**) of the representative case showed that the largest difference was mainly presented at air–tissue interfaces, air cavity, and bony areas. The plan on sCT_ICRU_ underestimated the doses at air cavity and air–tissue interfaces. However, the dose distribution on sCT_tailor_ slightly overestimated the dose to air cavity and air–tissue interfaces, but the dose difference near the tumor target was clearly decreased.

This study also proved that the bulk rED-assigned sCT guarantees clinically acceptable dosimetric accuracy for NPC patients under the 1.5 T magnetic field. In particular, assigning patient-specific rED values to sCT improves the dose calculation accuracy compared with using ICRU report 46 values. This study paves the way to a clinical implementation of the bulk sCT in online adaptive MR-guided radiotherapy for NPC patients.

However, there are also some potential limitations in this study. Firstly, the bulk sCT generation is highly dependent on the image registration process. Even though the MRI and CT images for each patient were acquired on the same day with the same setup procedure (patient-specific polyurethane foam immobilization devices and head–neck–shoulder immobilization mask), and rotations were also included in the rigid registration process, some errors still can be introduced. Besides that, the evaluation standard of registration quality is somehow subjective. In particular, the neck region cannot be perfectly matched due to the changes in neck flexion between different scans. The differences in anatomy changes during different image acquisitions can also introduce errors when comparing the sCT with the original CT. Nowadays, the bulk rED assignment is the only available method in the Unity Monaco system. With the development of advanced atlas-based, voxel-based, deformable registration-based, and deep learning-based sCT generation, this problem could be resolved with higher accuracy in the near future.

## Conclusion

This study demonstrated that the bulk rED-assigned sCT by adopting the patient-specific bulk rED values guarantees a clinically acceptable level of dose calculation accuracy for NPC patients in the presence of a 1.5 T magnetic field, making this approach suitable for online adaptive MR-guided radiotherapy for NPC patients.

## Data Availability Statement

The original contributions presented in the study are included in the article/supplementary material. Further inquiries can be directed to the corresponding author.

## Ethics Statement

The studies involving human participants were reviewed and approved by Sun Yat-sen University Cancer Center Ethics Committee (approval no. C2019-006-01). The patients/participants provided their written informed consent to participate in this study.

## Author Contributions

All authors contributed to the research. SD completed the treatment plans, performed the data analysis, and drafted the manuscript. XH and HL made contribution to the study’s conception and design. RL participated in the data analysis. YL and BW participated in the patients’ treatment and data interpretation. All authors contributed to the article and approved the submitted version.

## Funding

This work was supported by the National Natural Science Foundation of China (No. 11805292), the Guangdong Basic and Applied Basic Research Guangdong-Guangzhou Joint Youth Fund (No. 2021A1515110642), the Tumor Precision Radiotherapy Spark Program Clinical Research Fund Project (No. HDRS2020030205), and the Tumor Precision Radiotherapy Summit Program Clinical Research Fund Project (No. 2021-DF-009).

## Conflict of Interest

The authors declare that the research was conducted in the absence of any commercial or financial relationships that could be construed as a potential conflict of interest.

## Publisher’s Note

All claims expressed in this article are solely those of the authors and do not necessarily represent those of their affiliated organizations, or those of the publisher, the editors and the reviewers. Any product that may be evaluated in this article, or claim that may be made by its manufacturer, is not guaranteed or endorsed by the publisher.
